# Observations on the Employment of Compression in Aneurism

**Published:** 1846-12

**Authors:** O’Bryen Bellingham

**Affiliations:** The Medical Officers of St. Vincent’s Hospital


### RECORD OF MEDICAL SCIENCE.

*Observations on the employment of compression in Aneurism.* By O'Bryen Bellingham, M. D., F. R. C. S. I., one of the Medical Officers of St. Vincent's Hospital.—Throughout the period we have been considering, the names of but few British surgeons appear in connection with improvements in the treatment of aneurism, and none in connection with the treatment of this disease by compression. In fact, all the instruments for the application of pressure (which have been described) and all the improvements made in this method of treating aneurism, originated with our continental neighbours, amongst whom the French and Italian schools rank foremost. We have now, however, arrived at a period in the history of anuerism of the large arteries at which a great revolution in its treatment was brought about; and at which British surgery took the lead in improving the treatment of this disease, which it has maintained down to the present day.

In the year 1785, Hunter's first operation for popliteal aneurism (according to what is called the modern method) was performed. The great superiority of this operation over that formerly in use can only be fully understood by those who have perused the accounts of the cruel, I might say, barbarous operations, practiced previous to that period—operations which were not only painful and protracted, but almost always fatal to life; and which, if the patient escaped after the tedious suppuration which necessarily followed, left behind a contracted state of the joint and permanent lameness. Those who have had the opportunity of dissecting cases of the kind, can have but a faint idea of the difficulty which must have attended the application of a ligature, in the living subject, to the artery above and below the sac in a large aneurism seated in the ham, and of the tedious dissection which must have been necessary to accomplish it. Nevertheless, it was not until many years subsequently that the Hunterian method entirely supplanted the old operation : the latter was performed by Boyer so recently as in 1809. Writing in the year 1818, he observes, "in certain case sof popliteal aneurism the old operation is preferable to the Hunterian ;" and if the aneurism is seated in the middle of the thigh, or higher up, the old method is alone applicable, because the femoral artery would require to be tied above the origin of the profunda, "which would prodigiously diminish the resources of nature in keeping up the circulation in the limb."

The simplicity of the Hunterian operation, the facility with which it was performed, and the success which attended it when contrasted with the ancient method, led in a great measure to the discontinuance, by British surgeons, of compression in aneurism of the large arteries ; and to the almost entire discontinuance of the mode in which it had previously been used. We read no longer of cases of large popliteal aneurism treated by pressure upon the sac ; when pressure was used it was applied upon the artery between the aneurism and the heart—an improvement in the mode of its application which evidently sprung from a due appreciation of the principles upon which Hunter's operation was performed.

Nevertheless, owing to the failure of this operation in the hands of practitioners both in England and upon the continent, pressure was occasionally tried either previous to the performance of the operation, or in cases where, from the situation of the aneurism, the operation was deemed impracticable. Thus in two of the cases operated upon by Hunter, it is reported that "compression upon the femoral artery had been attempted previously, but the pain was so great that it could not be continued." In the ninth volume of the *London Medical Journal*, published in the year 1788, Mr. Ford has recorded a case of femoral aneurism which was situated too high up to admit either of amputation or of the application of a ligature, and in which "compression was endeavoured to be made upon the artery in the groin by means of an instrument somewhat resembling a steel truss ; but the pain it occasioned when the pressure was strong enough to restrain the pulsation of the tumour, soon obliged us to forego this experiment." The disease was left to itself, and a spontaneous cure eventually took place. Again, in the eighth volume of the *Medical and Physical Journal* for the year 1802, a case is given in which Mr. Blizard of the London Hospital attempted to effect a cure of popliteal aneurism by obliterating the femoral artery by pressure. The patient eventually came under the care of Sir A. Cooper, who has given the following description of the apparatus used :—"The points of support for this instrument were the outer part of the knee and the great trochanter, a piece of steel passing from the one to the other ; to the middle of this a semicircular piece of iron was fixed, which projected over the femoral artery, having a pad at its end moved by a screw, by turning which the artery was readily compressed, and the pulsation in the aneurism stopped, without any interruption to the circulation in the smaller vessels. But although this patient possessed unusual fortitude of mind and indifference to pain, he was incapable of supporting the pressure of the instrument longer than nine hours, and when it was loosened the pulsation in the tumour returned with unabated force. After a fair trial of this plan the patient left the hospital." He was admitted subsequently into Guy's Hospital, and operated upon with success by Sir A. Cooper.

The principle upon which surgeons applied pressure at this period was to endeavour to excite inflammation of the coats of the artery at the seat of its application ; they supposed that if by means of strong pressure the coats of an artery were brought into contact, and retained so, inflammation of the arterial tunics would follow, lymph would be thrown out, adhesion would take place between them, and the obliteration of the artery would be effected in the same manner as if a ligature had been applied. The first to endeavour to determine by direct experiment the effects of pressure upon the arterial tissue, was Mr. Freer of Birmingham, who, in the year 1806, performed a series of experiments upon animals with this object. He found that compression when applied with sufficient force to excite inflammation of the artery and the parts surrounding it, instead of causing lymph to be thrown out in the cavity of the artery, and adhesion of the sides of the vessel, (as was commonly supposed) occasioned an effusion of lymph about the artery and between its coats, by which its tube was so much compressed as to be impervious to the passage of blood. Two of his experiments we shall quote :

"*Experiment* 1.—December 5th, a tourniquet was applied, and a pad fixed upon the left radial artery of a horse, and a wide wooden splint was fixed on the opposite side of the limb to prevent the circulation being interrupted in the collateral vessels. On the evening of the same day the limb was much swelled, but retained its natural heat. On the evening of the 6th the tourniquet was removed until the middle of the following day, when it was again applied. By this time the œdema and swelling of the limb had greatly increased, and the heat was much more than natural. The pressure was removed about eight o'clock A. M., December 9th, and the animal was killed at two the same day."

"Upon dissecting, a great effusion of serum was found in the cellular membrane from the place where the tourniquet was applied to the hoof. There was also a small effusion above the tourniquet. There was great inflammation of the cellular membrane, fascia and muscles which lay under the pad of the tourniquet. The artery lay embedded in a quantity of coagulable lymph, effused into the cellular substance surrounding the vessel, and externally it appeared inflamed. The upper part of the artery exhibited no signs of disease : but when an incision was made along it, an effusion of lymph was found in its internal coats, which swelled them to that degree as to obliterate the cavity by pressing its sides in contact with each other for the space of an inch and a half.

"*Experiment* 2.—A tourniquet was applied, in the same manner as in the former experiment, to the radial artery of a horse, on the 16th of December. The following day the limb became œdematous: at night the tourniquet was a little slackened, but on the morning of the 18th it was again tightened, and continued so until the 20th, when the tourniquet was removed. On the 22nd the animal was killed.

"Upon dissection, there was not so much exudation of lymph below the compression, as in the former experiment. Around the artery there was much inflammation, but no inflammation of the artery itself. For two inches above and below the pad, it was so much diminished in its cavity as with much difficulty to admit of the introduction of a bristle, and immediately under the pad, a small plug of dark-coloured coagulum was formed, below which was a considerable protuberance of the coats of the vessel impeding the passage of the blood. The obstruction was farther increased by a great thickening of the coats of the artery, which had become almost of a cartilaginous texture."

The work in which Mr. Freer's experiments are recorded was published at Birmingham in the year 1807. In it he has given the details of two cases of aneurism, one of the femoral, the other of the popliteal artery, which were cured by compression of the entire limb by bandages and compresses. Neither of the cases were treated by Mr. Freer, and as this method of applying pressure has already been fully illustrated, it will only be necessary to quote one of them here.

"The patient, a guard of a mail coach, aged 35, ascribes the disease to a sudden jerk which he received in jumping off the coach. The knee became generally swollen, and a small pulsating tumour was felt in the ham, which gradually increased and in a few weeks attained the size of a large orange, pulsating violently, and producing much pain in the part, though the limb itself was benumbed. The compression was made by means of a roller carried from the toes up the leg and over the tumour. A small pad of linen was then applied to that part of the artery where it passes round the inner side of the thigh to get at the ham. The compress was then firmly bound down by the remaining turns of the roller, which was continued up the thigh. During the application of the roller, the lower limb was affected with agonizing and almost intolerable pain. It was, however, after one of these urgent paroxysms that the pulsation in the tumour was found to have ceased. The roller was continued for some weeks, during which the tumour considerably diminished. He was seen two year afterwards and experienced no inconvenience except a little lameness."

Mr. Freer is an advocate for a trial of compression in all cases of aneurism which admit of its application. "In the cases where compression has been hitherto used, (he observes) it has been upon the tumour itself; and although this mode of application has frequently been attended with success, it is by no means so likely to answer the intention of uniting the sides of the vessel as when used on the sound part of the artery. From the result of the experiments upon the radial artery of the horse, I should recommend the pressure to be applied on the extremities, either by the assistance of Senffio's instrument, or in the following manner.

"Apply a bandage moderately tight from one extremity of the limb to the other, and place a pad upon the artery a few inches above the tumour; then, with a common tourniquet surrounding the limb, let the screw be fixed upon the pad, having previously secured the whole limb from the action of the instrument by a piece of board wider than the limb itself, by which means the artery only will be compressed when the screw is tightened, the tourniquet should then be twisted till the pulsation in the tumour ceases. In a few hours, as by experiment in the horse, the limb will become œdematous and swelled; the tourniquet may then be removed, and no stronger pressure will be required than can easily be made with the pad and roller. The irritation produced by this mode of pressure excites that degree of inflammation of the artery which deposits coagulable lymph in the coats of the vessel, thickens them, diminishes the cavity, and eventually obstructs the passage of the blood."

The earliest examples of popliteal anuerism successfully treated by compression upon the artery above the tumour were recorded in the year 1810. The first is noticed by Pelletan in his Clinique Chirurgicale, published in that year, but the details are given very briefly. This case is related more at length by Richerand in the article Aneurysme in the second volume of the Dict. des Science Médicales, published in 1812; and still more fully by Boyer in the second volume of his Traité des Maladies Chirurgicales, published in 1818.*

The patient was a grocer living in one of the streets of the Isle St. Louis, Paris. The aneurism was seated in the ham, was of moderate size, and had a strong pulsation; there was no swelling of the limb. In consultation with Deschamps, Pelletan, Dubois and Boyer, compression upon the artery above the tumour was recommended, because, as the aneurism was neither of long standing nor of large size, there was no immediate necessity for an operation; besides, supposing that compression failed, it would be likely rather to favour the success of a subsequent operation. The patient having been made acquainted with the decision, was persuaded by a locksmith of his acquaintance to employ an instrument, which the father of the latter had invented, and had used with success upon himself when labouring under a similar disease. This Boyer states to have been

* With the exception of this case, the editor of the *Dublin Quarterly Journal of Medicine* (in his observations on the history of the cure of popliteal aneurism by compression, to which I have before had occasion to allude,) has overlooked all the examples which follow the successful employment of this method of treatment.

a kind of tourniquet of a rather ingenious construction; Richerand describes it as a modification of a truss, with a screw and a pad attached. M. Eschard, who had the charge of the patient, applied the compress upon the femoral artery, just above its passage through the tendinous canal in the triceps muscle. At first the pain it occasioned was so great that it had frequently to be relaxed; but by degrees the patient became accustomed to it, and at the end of eleven months, during which he had remained perfectly at rest, restricted himself to a low diet, with a bleeding once a month, the aneurism ceased to pulsate, the tumour became small and hard, and ultimately entirely disappeared.

The second case occurred in the practice of Dubois, and the patient was presented to the Faculté de Médecine at a meeting held March 29th, 1810. The case is recorded in the seeond volume of the Bulletins de la Faculté de Médecine.

The patient, a carter, aged 27, began to complain three months previously of pain and lameness in the left lower extremity, which became so severe in January that he was obliged to give up his occupation; at this time he first perceived a pulsating tumour in the popliteal space. By the 5th of February the aneurism had acquired the size of a large pigeon's egg, it pulsated strongly, and the pulsation could be checked by pressure upon the femoral artery; the colour of the skin was unchanged. The patient could neither walk nor stand without much pain, which extended from the ham to the back of the leg and the outside of the foot.

M. Dubois having determined to try the effect of compression upon the artery in the middle of the thigh, a preparatory bandage was applied over the tumour until the 24th of February, which produced no other change than a light diminution in its pulsation. On the 25th an apparatus, constructed expressly for the purpose, was applied: this consisted of a semicircle of iron, both extremities of which terminated in pads; one was applied upon the exterior of the thigh, the other upon its internal surface exactly opposite; to the latter a screw was attached, by turning which pressure upon the artery was made. On the 28th of February the pressure was increased; by the 2nd of March, the pulsation in the tumnur was perceptibly diminished. On the 7th, still stronger pressure was made, so as to check altogether the pulsation in the aneurism. Towards evening, the pressure became so painful that the patient removed the apparatus, when he found that the pulsation had ceased. The apparatus was replaced, and by the 12th, the tumour had become hard and small. On the 17th, it was removed altogether, but the patient was not permitted to leave his bed for some days longer. On the 29th, he was presented to the Faculté de Médecine perfectly cured.

The instrument employed in this case would appear from the description to have been very nearly similar to those recently used for the same purpose; it evidently served as the model of the compressor which bears Dupuytren's name.

On the 22nd of November, in the same year (1810), Viricel, surgeon to the Hótel Dieu de Lyon, communicated to the Faculté de

Médecine two cases of aneurism cured by compression; the details are not, however, given, nor is it even stated where the aneurisms were seated; but it is probable that they were cases of popliteal aneurism, because Lisfranc in his work on Aneurism mentions that Viricel had succeeded in curing more than one case of popliteal aneurism by pressure upon the artery above the tumour.

In the year 1811, the subject proposed by the Royal College of Surgeons of London for the Jacksonian prize was—"Wounds and Diseases of Arteries and Veins." Mr. Hodgson was the successful competitor, and his essay formed the groundwork of the valuable treatise upon aneurism which he afterwards published. In a former year the proposal of a similar subject by the Societé de Mèdecine of Paris, for their prize, led to the publication of Scarpa's work. From the year 1810, however, down to 1818, no cases of femoral or of popliteal aneurism have been recorded, in which pressure was successfully used; although this proceeding was occasionally had recourse to previous to the performance of the operation. Thus, in the seventh volume of the Medico-Chirurgical Transactions, in a paper upon aneurism by Sir Philip Crampton, a case is given in which compression by an instrument, similar to that used by Sir W. Blizard, was tried by Mr. Adrain, "but by no force that could be borne was he able to stop the pulsation at the ham;" the attempt was therefore abandoned.

Mr. Hodgson states that "in the only instance in which he has known compression to be used, the patient insisted on the removal of the instrument in less than an hour after its application; the pain which was produced by continued pressure was insupportable." In a note he adds, that he was informed by a friend of another case of popliteal anuerism, in which pressure could not be supported longer than two hours. "The compression of the femoral artery was effected by means of a tourniquet, a broad piece of wood being placed upon the opposite side of the limb, so as in some degree to prevent pressure upon the collateral vessels."

In the thirteenth volume of the *Edinburgh Medical and Surgical Journal*, Dr. A. Robertson has given a figure and description of an instrument which he used in one case of femoral aneurism. This consisted of a flat splint which was placed in an oblique direction under the hip, having a semicircular piece of iron passing over the thigh, which was attached at each extremity to the back of the splint. This was pierced for a screw, which terminated in a button; a graduated compress was laid upon the artery, and the button of the screw upon it; by turning the screw, the circulation of the artery was cheeked. The instrument was kept in its place by two or three turns of a roller carried round the body. The patient was prepared by bleeding and purgatives; the compress was laid upon the artery as it passes under Poupart's ligament, and compression made so as completely to check the pulsation in the aneurism. "The instrument remained on the limb for forty-eight hours, (with only two or three intervals of a few minutes) when the pain from the continued pressure became so excessive that he was unable to bear it longer."

At a meeting of the Faculté de Médecine, held on the 5th of November, 1818, Dupuytren presented a patient who had been cured of popliteal aneurism by pressure upon the artery in the middle of the thigh, by means of the instrument which bears his name; at the same time he stated that this was the second case in which he had employed pressure successfully in popliteal aneurism.*

The patient was a Pole, aged 30; the aneurism, which was seated in the ham, was of several years' standing, of the size of a turkey's egg, and was accompanied by very severe pain, which prevented the patient using the limb, and the motion of the joints were lost. Dupuytren proposed the operation, but the patient refused to submit to it. The compressing instrument was then applied upon the artery in the middle of the thigh, the patient was instructed how to use it, and was directed to relax the pressure when ever the pain became very severe; ice at the same time was applied to the tumour. About the 5th or 6th day the pain subsided, the sensibility of the limb returned, and the patient was able to move the joint a little; the aneurismal tumour had diminished in size, and on removing the instrument the pulsation of the aneurism was found to be less strong. On the 20th day the pulsation in the ham being no longer felt on the discontinuance of the pressure, the patient removed the instrument. A month afterwards he was examined by Dupuytren, the aneurism was perfectly cured, he had good use of the limb, and nothing remained in the site of the tumour but a hard knot, the size of a pigeon's egg, which diminished daily.

The instrument employed in the foregoing case goes under the name of Dupuytren's compressor. It consists of a semicircle of solid steel, having at one extremity a large concave cushion adapted to the posterior part of the limb; and at the other extremity a pad, which, by means of a spiral spring and screw, can be approximated to it. The pad is applied upon the artery we intend to compress, and by turning the screw pressure can be made to the requisite degree, the cushion affording the counter-pressure. Thus the compression is limited to two points upon the opposite side of the limb, and the main artery of the limb can be commanded without interrupting the current in the collateral vessels or superficial viens. Dupuytren was in the habit of employing this instrument in amputation in place of the tourniquet.

In the second volume of Boyer's Treatise on Surgery, published in the year 1818, we find another example of popliteal aneurism, in which compression was employed with success; Boyer had treated

* The editor of the *Dublin Quarterly Journal of Medicine*, with his usual accuracy, observes, in reference to the employment of compression in aneurism by Dupuytren about the year 1817, the Baron Dupuytren is *said* to have cured a case of popliteal aneurism by pressure; but Mr. Adams, who witnessed it, informs us that it did not turn out successfully.

the case many years previously, but it was then for the first time published.

"The patient, a boatman, aged 44, was admitted into La Charité on the 1st of February, 1805. The aneurism was seated in the right ham, had followed a sudden extension of the limb, and was of three months' duration. The tumour was about the size of an egg, was circumscribed, soft, pulsated strongly, and was partly reducible by pressure; compression upon the femoral artery checked its pulsation, and diminished its size. The limb was slightly swollen, but this disappeared after a few days rest.

Compression upon the femoral artery immediately above where the vessel passes through the tendinous canal in the triceps muscle was commenced, "the compressing instrument of Hunter" being employed; the pressure at first was very moderate, and the patient was sufficiently docile; but in the course of two or three months, much more firm pressure being used, the foot and thigh swelled, the patient complained of a sense of weight in the limb, and loosened the apparatus himself. The compression was then only used at intervals; after a time, however, he supported it better, and by the month of September, 1806, (when Boyer was obliged to leave Paris to accompany the Emperor in the Prussian campaign,) the tumour felt solid, had diminished in size, and the pulsation was less evident.

The pressure having been now continued for twenty months, Deschamps proposed the operation, and the patient gave his consent, but asked leave to return home for a few days, in order to arrange some matters. He left the hospital on the 2d of October, and returned in fifteen days; although he had been obliged to walk much in the interval, the aneurism had not increased in size, and its pulsation was no stronger than before. Deschamps therefore resolved to wait a little longer, and the compressing instrument was again applied. On the tenth day after its reapplication the patient stated, that on removing it, pulsation was no longer to be felt in the tumour; nevertheless, the compression was continued for eight days longer, when the patient was permitted to move about: the tumour became harder, and diminished daily, and he left the hospital on the 30th of November. He was seen frequently afterwards; nothing remained of the aneurismal swelling but a hard cord in the centre of the ham; the patient had perfect use of the limb.

In the ninth volume of the Medico-Chirurgical Transactions, published in the same year, Dr. Albers of Bremen, has reported a case of inguinal aneurism cured after the use of compression. In this case the pressure was applied directly upon the aneurismal sac, and the instrument used was similar to that already described as having been figured by Heister. "It consisted of a cushion fastened to a strap, which was buckled round the body. On the lower and inner side of the cushion there was also a strap, which was fastened round the thigh by means of a buckle. The cushion itself consisted of two iron pieces: the uppermost had the form of a common cushion, and was externally covered with leather: the lower piece was round and covered below with strong cloth, and above with leather. It was connected with the upper piece by a screw, by the operation of which its pressure on the tumour could be increased or diminished at pleasure."

The foregoing are the only examples of the successful employment of compression in aneurism of the large arteries which, down to this period, have been recorded. The latter, however, cannot properly come under this head, as the direct pressure upon the tumour appears to have caused inflammation in the sac and parts about it, which led to the obliteration of the artery above it. The cases in which compression is reported to have failed are still more numerous; Boyer has given the details of four in which he tried it, and Dupuytren two, in which this method was equally used without success.

In the first of Boyer's cases, the aneurism which was seated in the ham, occurred in a female. Compression was applied to the artery in the thigh by means of "Hunter's apparatus." On the 30th day although the pressure was not sufficient to check completely the pulsation in the aneurism, the patient complained much of the pain. On the 50th day the pressure had caused an eschar at the part, and the instrument had to be removed. In about three weeks it was re-applied a little higher up, and continued for three months, when the same cause obliged it to be again removed. It was tried a third time, but an eschar having again formed, it was abandoned, and the operation of laying open the sac performed with success.

In the second case pressure was applied by means of compresses and bandages, but the patient suffered so much pain that it had to be given up, and the same operation was performed with success.

The third case was also one of popliteal aneurism; the patient was admitted into hospital in March, 1809. The pressure was applied upon the artery in the thigh by the same instrument which had been used on a former occasion. It was continued for three months, the site of its application being occasionly changed, and its employment occasionally intermitted. At the end of this period the pressure was abandoned, although it is stated that the pulsation of the internal superior articular artery had become very perceptible; the operation of laying open the sac was then performed, and the patient died.

In the fourth case reported by Boyer, the patient had an aneurism in the ham, and another a little below Poupart's ligament upon the same side. On admission into La Charité in February, 1816, the leg was in a state of gangrene, and the limb was amputated above the knee. Nine days afterwards ice was applied to the other aneurism, combined with pressure upon the artery as it passes under Poupart's ligament, which, although used imperfectly, occasioned in the space of four days a diminution in the size of the aneurism, and lessened its pulsation; but the stump swelled, and an abscess formed in it, which did not, however, materially interfere with its healing, which took place at the end of fifty days.

The ice and pressure were continued, and on the 8th of May graduated compresses and a bandage were applied along the stump, so as to check the pulsation of the aneurism, but the patient left the hospital a few days afterwards. At this time the aneurism was much smaller and harder, and its pulsation much less strong than upon the patient's admission. He continued to employ the bandage and compresses until the month of March, 1818, at which period it is stated the pulsation of the aueurism was feeble, but still perceptible.

At a meeting of the Faculté de Médicine, held in July, 1819, Dupuytren presented two patients upon whom compression had been tried by him, but without success; and on whom he had afterwards operated. In one of these cases the aneurism was seated high up in the femoral artery; he employed an instrument by which pressure could be made upon the artery at it passes over the ramus of the pubis, the counter-pressure being upon the sacrum; but as it was readily displaced he had another constructed somewhat upon the principle of a truss, which, from the description, resembled that figured by Heister. After a trial of ten days the patient refused to continue its use, although at this period "the aneurismal tumour had been reduced to two-thirds of its original volume, and the strength of its pulsation had sensibly diminished."

Dupuytren concluded from these and several other analogous cases, "that there are individuals who cannot bear compression, whilst there are others who support it without inconvenience, and that this is the only reason of the different results of compression as applied to the treatment of aneurism." *Dublin Med. Press.*

